# Supplementary material for: Antibiogram Profile of Acinetobacter baumannii Recovered from Selected Freshwater Resources in the Eastern Cape Province, South Africa
Source: Pathogens. 2021 Aug 31;10(9):1110. doi: 10.3390/pathogens10091110 (PMC8466806; doi:10.3390/pathogens10091110)
Supplement: Supplementary file 1 [file pathogens-10-01110-s001.zip › pathogens-1237561-Supplementay.pdf]

Supplementary Figures

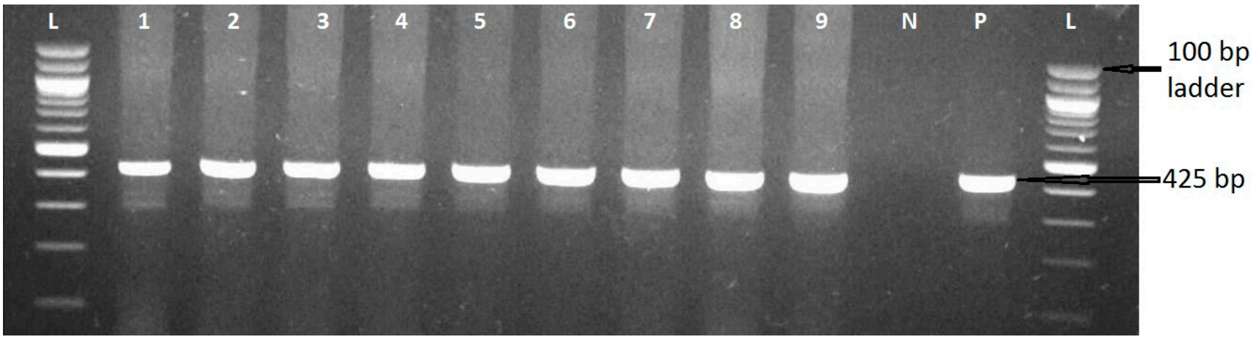

Figure S1: PCR analyses resolved by gel electrophoresis showing confirmed *Acinetobacter* spp. targeting the *recA* gene at 425 bp. L= DNA Ladder (100bp); Lane 1 to 9 = Selected *Acinetobacter* isolates; N= Negative control; P=Positive control (*A. baumannii,* DSM Number: 102929).

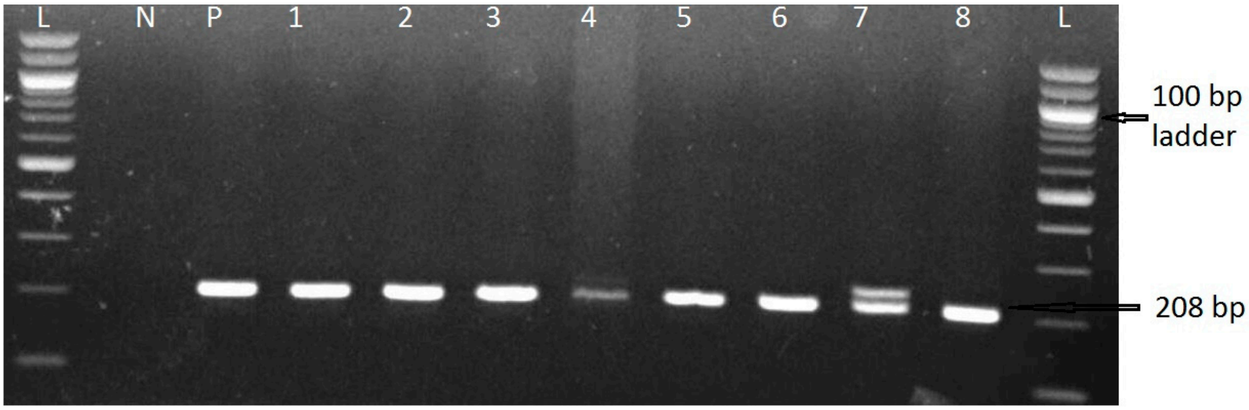

Figure S2: PCR analyses resolved by gel electrophoresis showing confirmed *A. baumannii* targeting the specific primer *Ab-ITS* gene at 208 bp. L= DNA Ladder (100bp); N= negative control, P= positive control (*A. baumannii,* DSM 102929); Lanes 1 to 8= selected *A. baumannii* samples.

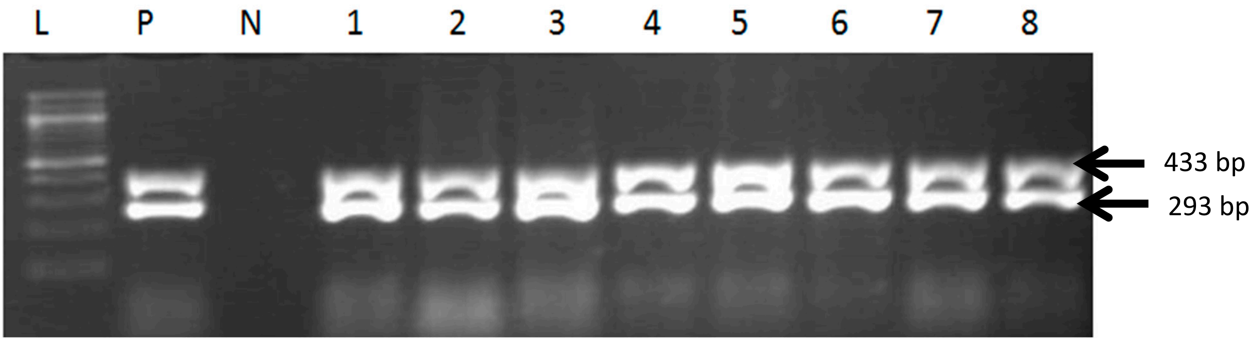

Figure S3: A gel electrophoresis showing sulfonamide resistance genes in *A. baumannii.* L= Ladder (100 bp); P= positive control, N= negative control, Lane 1 to 8 = Selected *A. baumannii* harbouring *sul1* (433 bp) and *sul2* (293 bp) genes.

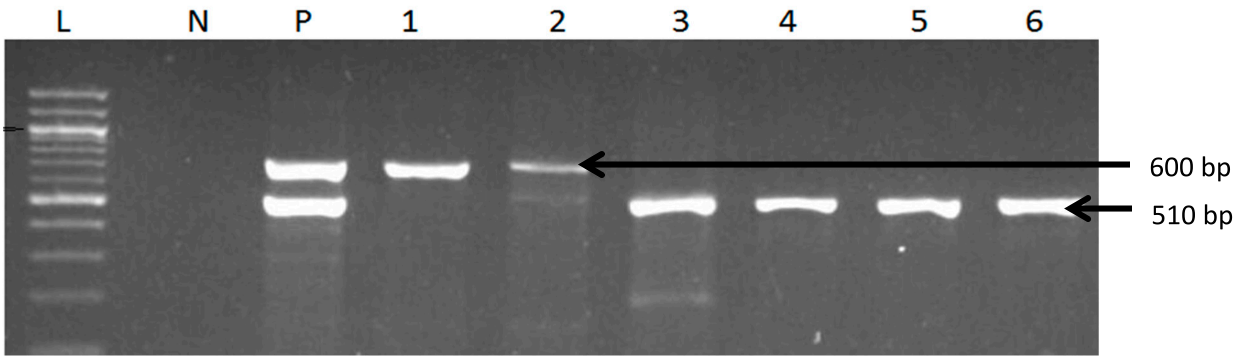

Figure S4: A gel electrophoresis showing aminoglycoside genes in *A. baumannii*. L= Ladder (100bp); P= positive control, N= negative control, Lane 1 and 2 = *apHA1* (600 bp) gene; lanes 3-6 = *apHA2* (510 bp) gene.

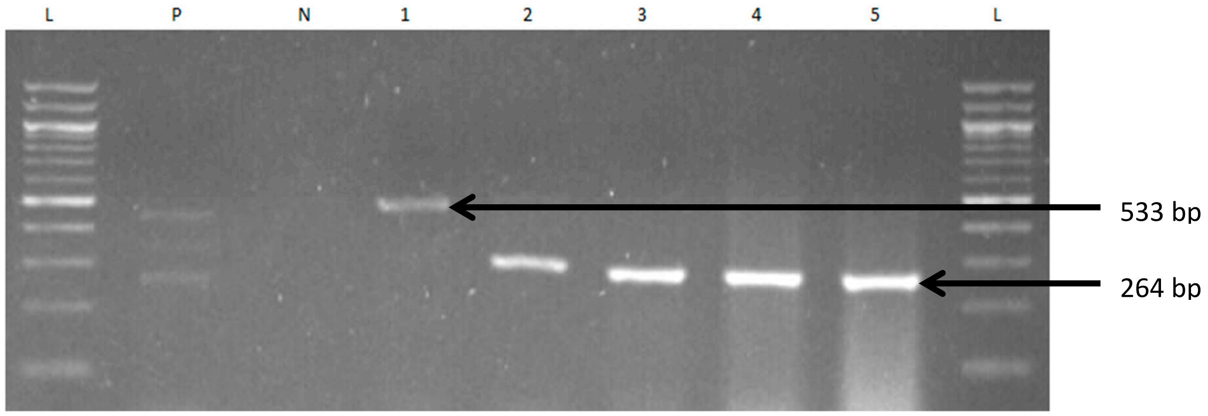

Figure S5: A gel electrophoresis showing Fluoroquinolones genes in *A. baumannii*. L= Ladder (100bp); P= positive control, N= negative control, Lane 1 = *qnrD* (533 bp) gene; lanes 2-5 = *qnrB* (264 bp) gene.

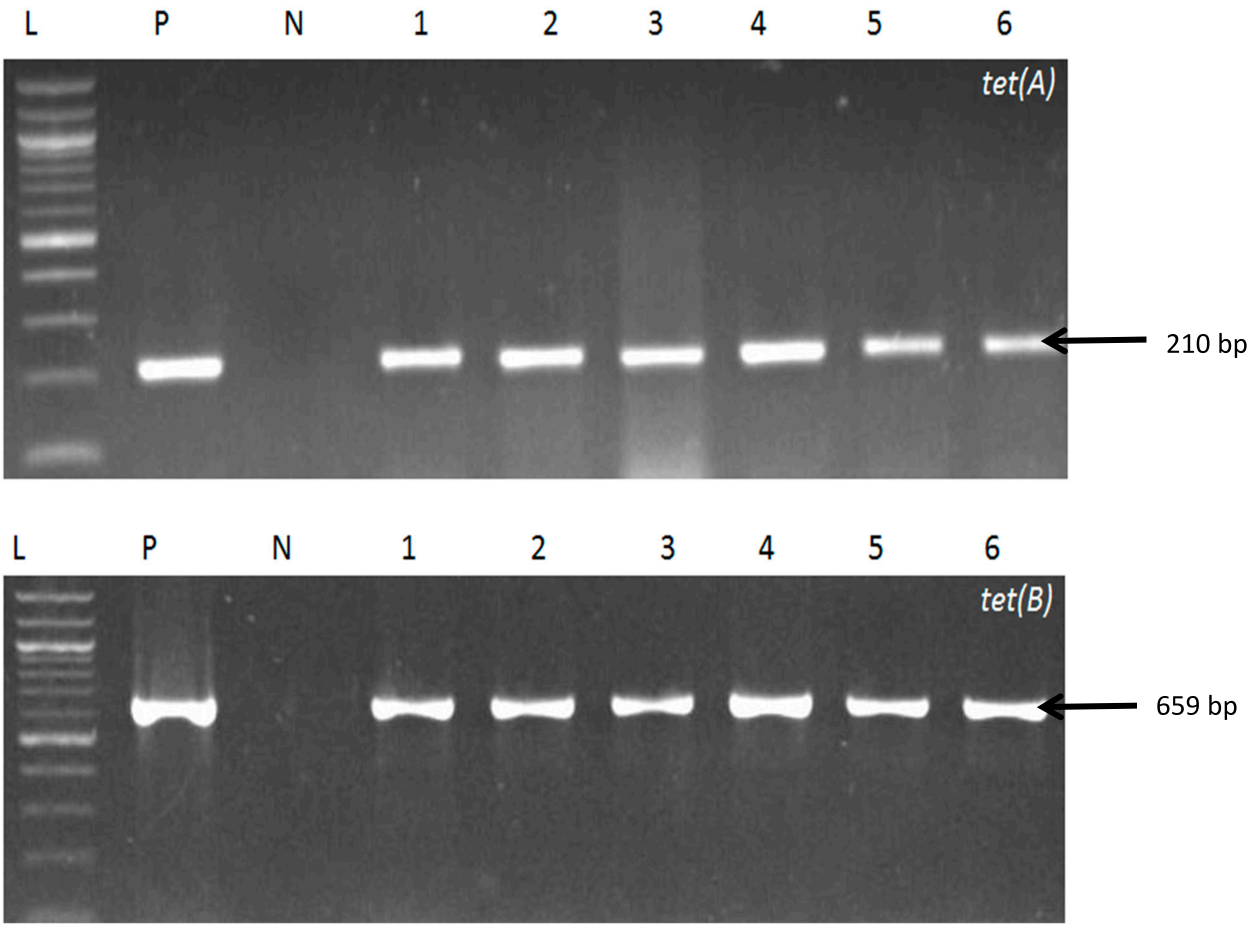

Figure S6: A gel electrophoresis showing Tetracycline genes *A. baumannii.* L= Ladder (100bp); P= positive control, N= negative control, Lane 1-6 = *tet*(*A*) (210 bp) and *tet*(*B*) (659 bp) genes respectively.

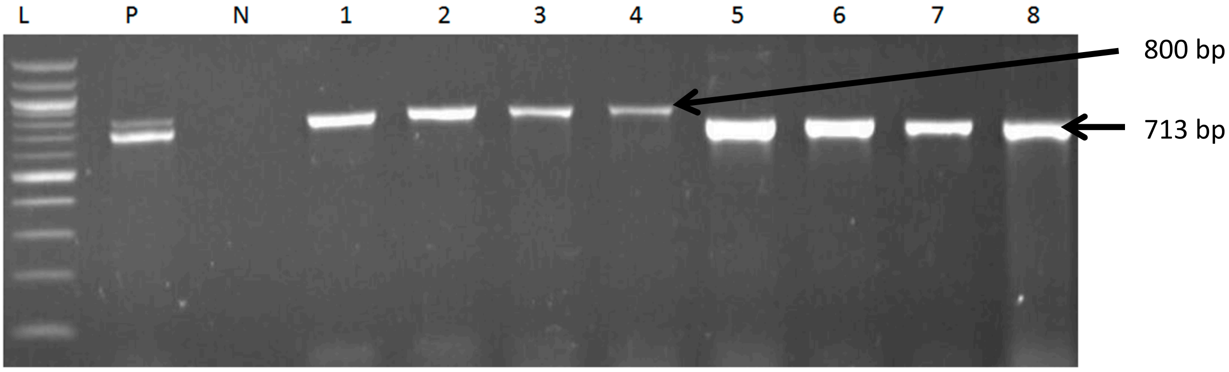

Figure S7A: A gel electrophoresis showing beta-lactamases genes in *A. baumannii.* L= Ladder (100bp); P= positive control, N= negative control, Lane 1-4 = *bla*$_{TEM}$ (800 bp) and 5-8= *bla*$_{SHV}$ (713 bp).

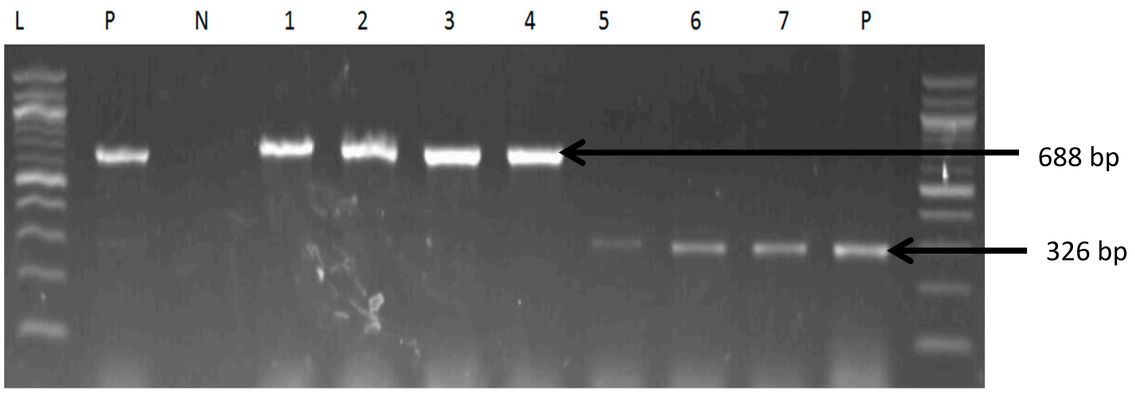

Figure S7B: A gel electrophoresis showing beta-lactamases genes in *A. baumannii*. L= Ladder (100bp), Lanes 1-4 = *bla*$_{CTX\text{-}M(1)}$ (688 bp), Lanes 5-7 = *bla*$_{CTX\text{-}M\text{-}8/\text{-}25}$ (326 bp).

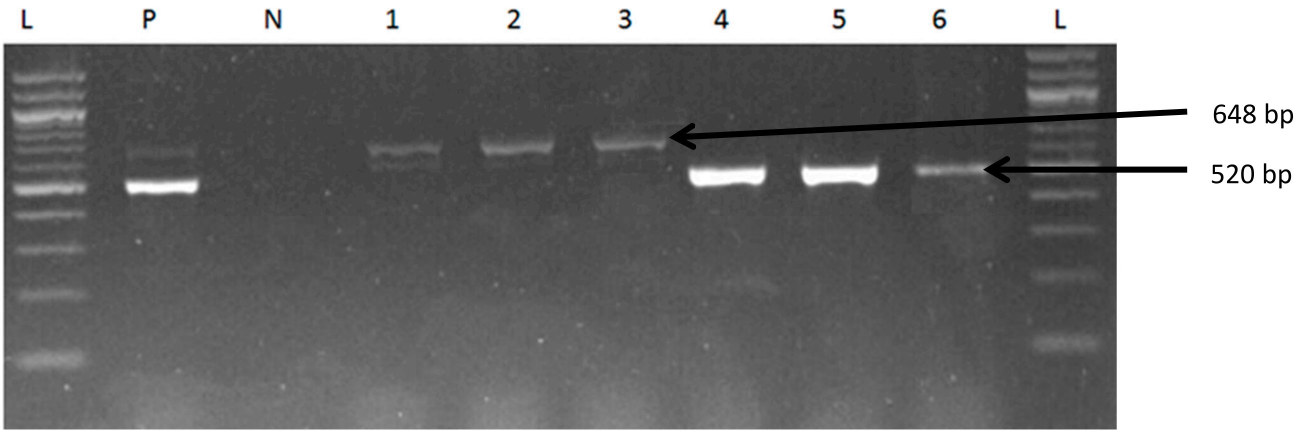

Figure S7C: A gel electrophoresis showing beta-lactamases genes in *A. baumannii*. Lane 1-3= *bla*$_{VEB}$ (648 bp) and Lane 4-6 = *bla*$_{PER}$ (520 bp) genes.

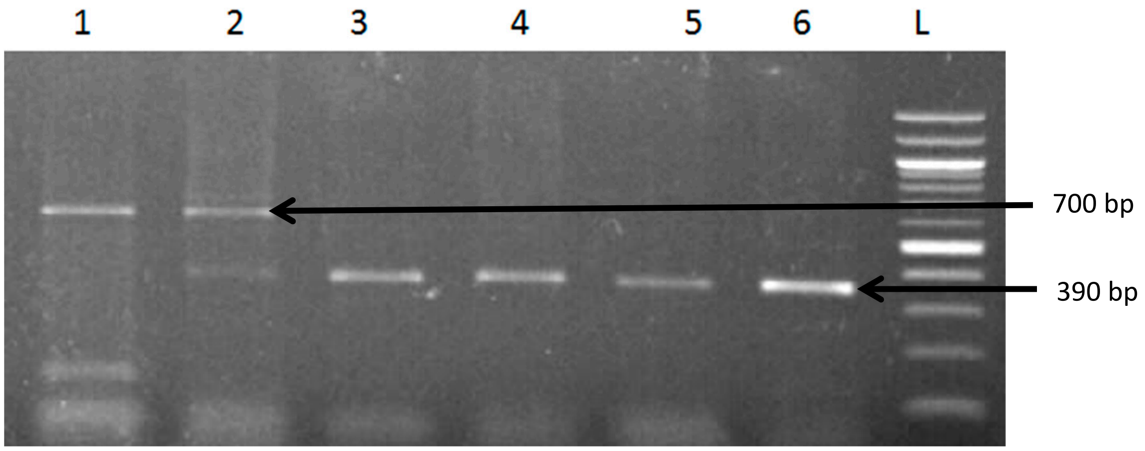

Figure S7D: A gel electrophoresis showing beta-lactamases genes in *A. baumannii*. L= Ladder (100bp), Lanes 1 and 2 = *bla*$_{KPC}$ (700 bp) and Lanes 3-6= *bla*$_{VIM}$ (390 bp). There were no positive and negative controls.

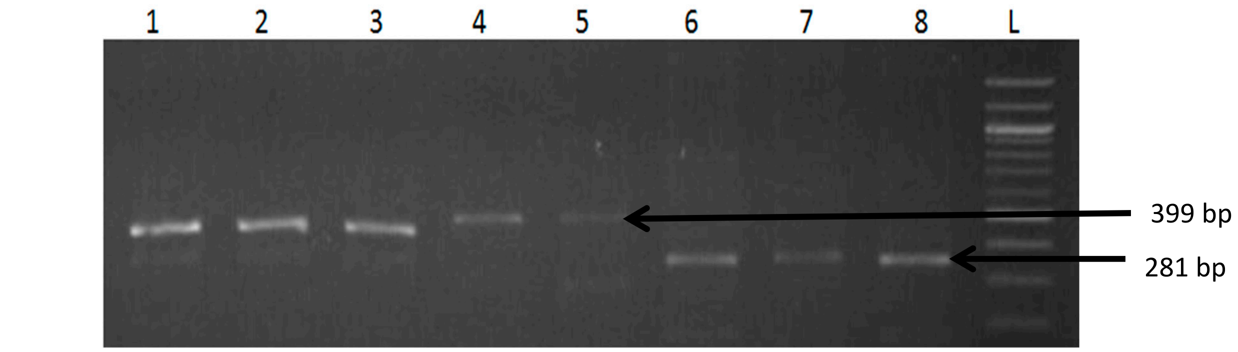

Figure S7E: A gel electrophoresis showing beta-lactamases genes in *A. baumannii*. L= Ladder (100bp); Lanes 1-5= *bla*$_{GES}$ (399 bp) and Lanes 6-8= *bla*$_{OXA\text{-}48\text{-}like}$ (281 bp). There were no positive and negative controls for both genes.

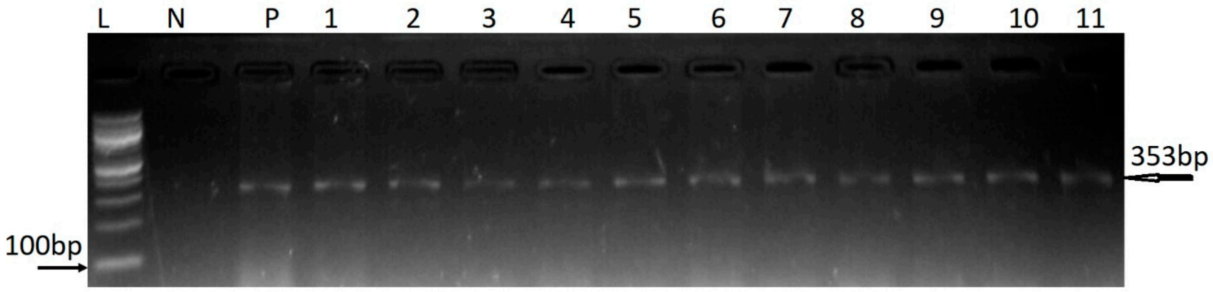

Figure S7F: A gel electrophoresis showing beta-lactamases genes in *A. baumannii*. L= Ladder (100bp); Lanes N = Negative control and Lanes P= Positive control. Lane 1-11= *bla*$_{OXA\text{-}51}$ (353bp).

Supplementary Tables

Table S1: Phenotypic antibiotic resistance of *A. baumannii* recovered from each of the site where that water samples were collected along Great Fish, Kieskamma and Tyhume River. S, R and I stand for susceptible, resistance and intermediate resistance respectively.

Site	Isolates code	Resistance Phenotypes										
		AK	CPM	CTX	CAZ	CIP	GM	IMI	MEM	PTZ	TET	SXT
TY1	1	S	S	S	S	S	S	S	S	S	S	S
TY1	20	S	S	R	S	S	S	S	R	S	S	S
TY2	22	S	S	I	S	S	S	S	S	S	S	S
TY2	23	S	S	I	S	S	S	S	S	S	S	S
TY2	26	S	S	S	S	S	S	S	S	S	S	S
TY2	28	S	S	I	S	S	S	S	S	S	S	S
TY2	38	S	S	I	S	I	S	S	S	S	S	R
TY3	43	S	S	I	S	S	S	S	S	S	S	S
TY3	44	S	S	I	S	S	S	S	S	S	S	S
TY3	49	S	S	I	S	S	S	S	S	S	S	S
TY3	52	S	S	I	S	S	S	S	S	S	S	S
TY3	53	S	S	I	S	S	S	S	S	S	S	S
TY3	55	S	S	I	I	S	S	S	S	S	S	S
TY4	63	S	S	I	I	S	S	S	S	I	S	S
TY4	64	S	S	R	S	I	S	S	S	S	S	S
TY4	68	S	S	I	S	S	S	S	S	S	I	S
TY4	69	S	S	I	S	S	S	S	S	S	S	S
TY4	72	S	S	I	I	S	S	S	S	I	S	S
TY4	75	S	S	I	S	S	S	S	S	S	S	S
TY4	76	S	S	I	S	S	S	S	S	S	S	S
TY5	80	S	S	S	S	S	S	S	S	S	S	S
TY5	81	S	S	I	S	I	S	S	S	S	S	S
TY5	83	S	S	I	S	S	S	S	S	S	S	R
TY5	84	S	S	S	S	S	S	S	S	S	S	S
TY5	85	S	S	I	S	S	S	S	S	S	S	S
TY5	87	S	S	I	S	S	S	S	S	S	S	S
TY5	88	S	S	S	S	S	S	S	S	S	S	S
TY5	90	S	S	S	S	S	S	S	S	S	S	S
TY5	91	S	S	I	S	S	S	S	S	S	S	S
TY5	92	S	S	I	S	S	S	S	S	S	S	R
TY5	93	S	S	I	S	S	S	S	S	S	S	S
TY5	99	S	S	I	S	S	S	S	S	S	S	S
KE1	106	S	S	I	S	S	S	S	S	S	S	R
KE1	107	S	S	I	S	S	S	S	S	S	S	R
KE4	127	S	S	I	S	S	S	S	S	S	S	S
GF2	153	S	S	I	S	S	S	S	S	S	S	R

GF2	154	S	S	I	S	S	S	S	S	S	S	S
GF2	156	S	S	I	S	S	S	S	S	S	I	R
GF2	159	S	S	R	S	I	S	S	I	I	I	R
GF3	167	S	S	I	S	S	S	S	S	S	I	S
GF3	170	S	S	S	S	S	S	S	S	S	S	S
GF4	174	S	S	I	S	S	S	S	S	S	S	R
GF4	176	S	S	I	S	S	S	S	S	S	S	S
GF5	179	S	S	I	S	S	S	S	S	I	I	R
GF1	187	S	S	I	S	S	S	S	S	S	S	R
GF1	188	S	I	R	I	I	S	S	S	I	S	S
GF1	195	S	S	I	I	S	S	S	S	I	S	S
GF1	198	S	S	I	S	S	S	S	S	S	S	S
GF2	208	S	S	I	S	S	S	S	S	S	S	S
GF2	209	S	S	R	S	I	S	S	S	S	S	S
GF2	213	S	S	I	S	S	I	S	S	R	S	S
GF2	214	S	S	I	S	S	S	S	S	S	S	S
GF2	216	S	S	I	S	S	S	S	S	S	S	S
GF3	227	S	S	S	S	S	S	S	S	S	S	S
GF4	232	S	S	I	S	R	S	S	S	S	S	R
GF4	235	S	S	I	S	S	S	S	S	S	S	S
GF4	236	S	S	I	S	S	S	S	S	R	S	S
GF4	242	S	S	I	S	S	S	S	S	S	S	I
GF4	243	S	S	I	S	S	S	S	S	S	S	S
GF4	245	S	S	I	S	I	S	S	S	S	S	S
GF4	246	S	S	I	I	I	S	S	S	I	S	S
GF4	247	S	S	I	S	S	S	S	S	S	S	S
GF4	249	S	S	I	S	S	S	S	S	S	S	S
GF4	250	S	S	I	S	S	S	S	S	S	S	S
GF5	256	S	S	I	S	I	S	S	S	S	S	S
TY3	272	S	S	I	S	S	S	S	S	S	S	S
TY3	273	S	S	I	S	I	S	S	S	S	S	S
TY3	274	S	S	I	S	I	S	S	S	I	S	S
TY3	278	S	S	I	S	I	S	S	S	S	S	S
TY3	279	S	S	I	S	S	S	S	S	S	S	S
TY3	282	S	S	I	S	S	S	S	S	S	S	S
TY3	283	S	S	I	S	I	S	S	S	S	S	S
TY3	286	S	S	I	S	S	S	S	S	S	S	S
TY3	289	S	S	I	S	S	S	S	S	S	S	S
TY3	290	S	S	I	S	I	S	S	S	I	S	S
TY3	294	S	S	I	S	I	S	S	S	S	S	S
TY5	303	S	S	I	S	I	S	S	S	S	S	S
KE1	306	S	S	I	I	S	S	S	S	S	S	S
KE1	308	S	S	I	S	S	S	S	S	I	S	S

KE1	309	S	S	S	S	S	S	S	S	S	S	S
KE1	315	S	S	I	S	S	S	S	S	S	S	S
KE1	316	S	S	I	I	I	S	S	S	S	S	S
KE2	319	S	S	I	S	S	S	S	S	I	S	S
KE4	333	S	S	I	S	S	S	S	S	S	S	S
TY2	346	S	S	I	S	S	S	S	S	S	S	S
TY2	348	S	S	S	S	S	S	S	S	S	I	S
TY2	353	S	S	I	I	S	S	S	S	I	S	S
TY2	356	S	R	I	S	S	S	S	S	S	S	S
TY2	358	S	S	I	S	S	S	S	S	S	S	S
TY3	361	S	S	I	I	I	S	S	S	S	S	S
TY3	364	S	S	I	S	I	S	S	S	S	S	S
TY3	367	S	S	I	I	I	S	S	S	I	S	S
TY3	369	S	S	I	I	S	S	S	S	S	S	S
TY3	370	S	S	I	I	S	S	S	S	S	S	S
TY3	372	S	S	I	S	S	S	S	S	S	S	S
TY3	373	S	S	I	S	S	S	S	S	S	S	S
TY3	374	S	S	I	I	S	S	S	R	S	S	S
TY3	378	S	S	I	S	I	S	S	S	S	S	S
TY3	380	S	R	I	S	I	S	S	S	S	S	S
TY3	382	S	S	I	I	S	S	S	S	S	S	S
TY3	385	R	R	R	R	R	R	S	S	S	S	S
TY3	386	R	R	R	R	R	R	S	S	R	S	S
TY4	388	R	R	R	R	R	I	S	R	R	R	R
TY4	389	S	S	I	I	S	S	S	S	S	S	S
TY4	394	S	S	R	I	S	S	S	S	S	S	I
TY4	396	S	S	I	S	S	S	S	S	S	S	S
TY4	397	S	S	R	I	S	S	S	S	I	S	I
TY4	398	S	R	R	I	R	S	S	S	R	R	S
TY4	401	S	S	I	I	S	S	S	S	S	S	S
TY4	404	S	S	I	S	S	S	S	S	S	S	S
TY4	406	S	S	I	S	S	S	S	S	S	S	S
TY4	407	S	S	I	I	I	S	S	S	I	S	I
TY4	409	S	S	R	R	S	S	S	S	I	S	S
TY4	413	S	R	I	I	S	S	S	I	S	S	S
TY4	415	S	S	R	I	I	S	S	R	S	S	I
TY4	422	R	S	R	I	S	S	S	S	R	S	I
TY5	431	S	S	R	I	S	S	S	S	R	S	S
TY5	432	S	S	I	S	I	S	S	S	S	S	S
TY5	433	S	S	I	I	S	S	S	S	S	S	S
TY5	434	S	S	R	R	S	S	S	S	I	S	S
TY5	436	S	S	I	I	S	S	S	S	S	I	S
GF1	441	S	S	I	S	S	S	S	S	S	S	S

GF2	444	S	S	I	S	S	S	S	S	S	S	S
GF2	449	S	S	I	S	S	S	S	S	R	S	S
GF2	450	S	S	I	I	R	S	S	S	S	S	S
GF3	454	S	S	I	S	S	S	S	S	R	S	S
GF3	455	S	S	I	S	S	S	S	S	R	I	S
GF3	456	S	S	I	I	S	S	S	S	I	S	I
GF4	459	S	S	I	S	S	S	S	S	I	S	R
GF4	460	S	S	I	S	S	S	S	S	S	S	I
GF4	461	S	S	R	I	S	S	S	S	I	I	R
GF5	463	S	S	R	I	S	S	S	S	I	I	R
GF5	464	S	S	R	I	S	S	S	S	S	S	R
GF5	465	S	S	R	I	S	S	S	S	I	S	R
GF5	466	S	R	I	S	S	S	S	S	S	S	I
GF5	467	S	S	I	S	S	S	S	S	S	S	S
GF5	468	S	S	R	I	S	S	S	S	I	S	I
GF5	469	S	S	R	I	R	S	S	I	I	S	I
GF5	471	S	S	R	S	R	S	S	S	I	S	I
GF5	473	S	I	R	I	R	S	S	S	R	S	I
KE2	475	S	S	I	I	I	S	S	S	I	S	S
KE2	477	S	S	R	I	I	S	S	S	I	S	S
KE2	481	S	I	R	I	I	S	S	S	S	S	R
KE2	482	S	S	I	I	I	S	S	S	R	S	S
KE2	484	S	S	R	I	I	S	S	S	I	R	S
KE2	485	S	S	R	I	I	S	S	S	I	R	I
KE2	486	S	S	I	I	I	S	S	S	I	S	S
KE2	489	S	S	I	S	S	S	S	S	R	S	S
KE2	490	S	S	R	I	I	S	S	S	I	S	I
KE3	494	S	R	I	S	I	S	S	S	S	S	S
KE3	496	S	S	S	S	S	S	S	S	S	S	S
KE3	497	S	S	I	S	S	S	S	S	S	S	S
KE3	499	S	I	I	I	S	S	S	R	S	S	S
KE4	501	S	S	I	S	S	S	S	S	S	S	S
KE5	504	S	S	I	S	S	S	S	S	S	S	S
TY2	505	S	S	I	S	S	S	S	S	S	S	S
TY2	509	S	S	I	S	I	S	S	S	S	S	S
TY2	510	S	S	I	S	I	S	S	S	S	S	S
TY2	511	S	S	S	S	I	S	S	S	S	S	S
TY3	513	S	S	I	I	S	S	S	S	R	R	R
TY3	514	S	S	I	I	S	S	S	S	S	S	S
TY3	515	S	S	I	I	S	S	S	S	S	S	I
TY3	516	R	S	I	I	S	S	S	S	S	S	S
TY4	519	S	S	I	S	S	S	S	S	S	S	S
TY4	520	R	S	I	I	R	S	S	S	I	S	I

TY5	523	S	S	I	S	S	S	S	S	S	S	S
TY5	524	S	S	I	S	S	S	S	S	S	S	S
TY5	527	S	S	I	S	S	S	S	S	S	S	S
TY5	528	S	S	I	S	S	S	S	S	S	S	S
TY5	529	S	R	R	R	S	S	S	S	I	S	S
TY5	531	S	R	R	R	S	S	S	S	I	R	S
KE2	536	S	R	R	R	I	R	S	I	R	S	R
KE2	537	S	R	I	R	S	R	S	S	S	R	R
KE2	538	S	S	I	R	R	R	I	I	R	R	R
KE2	539	S	R	R	R	S	R	S	S	I	I	R
KE2	540	S	S	I	S	R	R	I	R	R	R	R
KE2	541	S	S	I	S	R	S	S	I	R	R	R
KE2	542	S	S	I	S	R	S	S	S	I	R	R
KE3	543	S	S	I	S	S	S	S	S	S	S	S
KE3	544	R	S	I	S	S	S	S	S	S	S	S
KE3	545	S	S	I	S	S	S	S	S	S	S	S
KE3	546	S	S	R	S	R	S	S	S	S	S	S
KE3	547	S	S	R	R	R	R	S	S	S	R	R
KE3	548	S	S	I	S	S	S	S	S	S	S	S
KE3	549	S	S	I	S	S	S	S	R	R	S	S
GF1	556	S	S	I	S	S	S	S	I	I	S	R
GF1	557	S	S	I	S	S	S	S	S	S	I	S
GF1	559	S	S	I	S	S	S	S	S	S	I	S
GF2	560	S	S	I	S	S	S	S	S	R	S	S
GF3	564	S	S	I	S	S	S	S	S	S	S	S
GF3	565	S	S	I	S	S	S	S	S	I	S	S
GF3	567	S	S	I	S	S	S	S	S	S	S	S
GF3	568	S	S	I	S	S	S	S	S	S	S	S
GF3	569	S	S	I	S	S	S	S	S	S	S	S
GF4	570	S	S	S	S	S	S	S	S	S	S	S
GF4	571	S	S	S	S	S	S	S	S	S	S	S
GF4	573	S	S	S	S	S	S	S	S	S	S	S
GF4	574	S	S	I	S	S	S	S	S	S	I	S
GF5	575	S	S	I	S	S	S	S	S	S	S	S
GF5	576	S	S	I	S	S	S	S	S	S	S	S
GF5	577	S	S	I	S	S	S	S	S	S	S	S
GF5	578	S	S	I	S	S	S	S	S	S	S	S
GF5	580	S	S	I	S	S	S	S	S	S	S	S
GF5	582	S	S	I	S	S	S	S	S	S	S	S
KE1	583	S	S	I	S	S	S	S	S	S	S	S
KE1	586	S	S	I	S	S	S	S	S	I	S	S
KE2	591	S	R	R	R	R	R	S	I	R	R	R
KE2	592	S	R	R	R	R	R	S	S	R	R	R

KE2	593	S	R	R	R	R	R	S	I	R	R	R
KE2	595	R	R	R	R	R	R	I	I	R	R	R
KE2	596	S	R	R	R	R	R	S	I	R	R	R
KE5	622	S	I	R	R	R	R	S	S	S	R	I
GF1	625	S	S	I	S	S	S	S	S	S	S	S
GF2	630	S	S	R	R	R	R	S	S	I	R	I
GF4	636	S	S	I	S	S	I	S	S	I	S	R
GF4	637	S	S	I	S	S	S	S	S	I	S	S
GF4	638	S	S	R	R	R	R	S	S	R	R	I
GF4	639	S	S	I	S	S	S	S	S	S	I	I
GF4	640	S	S	I	S	I	I	S	S	S	S	S
GF4	642	S	S	I	R	R	S	S	S	I	S	S
GF4	643	S	I	R	R	R	R	S	S	I	R	I
GF4	645	S	S	I	S	S	S	S	S	S	S	S
GF5	647	S	S	I	S	S	S	S	S	S	S	S
GF5	648	S	S	I	S	S	S	S	S	S	S	S
GF5	649	S	S	I	S	S	S	S	S	S	S	S
GF5	650	S	S	I	S	S	S	S	S	S	S	S
GF5	651	S	S	I	S	S	S	S	S	S	S	S
GF5	652	S	S	I	S	S	S	S	S	S	S	S
TY2	658	S	S	I	S	S	S	S	S	S	S	S
TY2	659	S	S	I	S	S	S	S	S	S	S	S
TY5	673	S	S	I	S	S	S	S	S	S	S	S
TY5	674	S	S	R	S	S	S	S	S	S	S	S
TY5	680	S	S	I	S	S	S	S	S	S	S	S
GF2	691	S	S	S	S	S	S	S	S	S	S	S
GF3	692	S	S	I	S	S	S	S	S	S	S	S
GF4	695	S	S	I	S	S	S	S	S	S	S	S
GF4	696	S	S	I	S	S	S	S	S	S	S	S
GF4	697	S	S	I	R	S	S	S	S	S	S	S
GF4	698	S	S	I	I	S	S	S	S	S	I	S
GF4	699	S	S	I	S	S	S	S	S	S	S	S
GF4	700	S	S	I	S	S	S	S	S	S	S	S
GF4	701	S	S	I	S	S	S	S	S	S	S	S
GF4	704	S	S	S	S	S	S	S	S	S	S	S
GF4	705	S	S	I	S	S	S	S	S	S	S	S
GF4	706	S	S	I	S	S	S	S	S	S	S	S
GF4	707	S	S	I	S	S	S	S	S	S	I	S
GF4	708	S	S	S	S	S	S	S	S	S	S	S
TY1	712	S	R	R	R	S	S	S	S	S	R	S
TY1	713	S	S	S	S	S	S	S	S	S	S	S
TY2	715	S	S	S	S	S	S	S	S	S	S	S
TY2	716	S	S	S	S	S	S	S	S	S	S	S

TY2	717	S	S	S	S	S	S	S	S	S	S	S
TY2	718	S	S	S	S	S	S	S	S	S	S	S
TY2	719	S	S	S	S	S	S	S	S	S	S	S
TY2	720	S	R	R	R	S	S	S	S	R	R	S
TY2	723	S	S	S	S	S	S	S	S	S	S	S
TY2	724	S	S	S	S	S	S	S	S	S	S	S
TY3	725	S	S	S	S	S	S	S	S	S	S	S
TY3	726	S	S	I	S	S	S	S	S	S	S	S
TY3	727	S	S	S	S	S	S	S	S	S	S	S
TY3	728	S	S	S	S	S	S	S	S	S	S	S
TY4	729	S	S	S	S	S	S	S	S	S	S	S
TY4	730	S	S	S	S	S	S	S	S	S	S	S
TY5	734	S	S	S	S	S	S	S	S	S	S	S
TY5	735	S	S	S	S	I	S	S	S	S	I	S
TY5	736	S	I	S	S	S	S	S	S	S	S	S
TY5	738	S	S	S	S	S	S	S	S	S	S	R
KE1	739	S	S	I	I	S	S	S	S	S	S	S
KE1	740	S	S	S	S	S	S	S	S	S	S	S
KE1	741	S	S	S	S	S	S	S	S	S	S	S
KE3	756	S	S	R	R	R	R	S	S	S	R	R
TY1	757	S	S	I	I	I	I	S	S	S	S	R
TY1	758	S	S	S	R	S	S	S	S	S	S	S
TY2	761	S	S	S	S	S	S	S	S	S	S	S
TY2	765	S	S	I	S	S	S	S	S	S	S	R
TY4	771	S	S	S	S	S	S	S	S	S	S	S
TY4	772	S	S	S	S	S	S	S	S	S	S	S
GF1	779	S	S	I	S	S	S	S	S	S	S	S
GF2	781	S	S	I	S	S	S	S	S	I	S	R
GF2	782	S	S	I	S	S	S	S	S	S	S	S
GF2	783	S	I	I	S	S	S	S	S	S	R	R
GF2	786	S	S	I	S	S	S	S	S	S	S	S
GF2	789	S	S	I	S	S	S	S	S	S	S	S
GF3	791	S	S	I	S	S	S	S	S	S	I	R
GF3	793	S	S	I	S	S	S	S	S	S	S	S
GF4	795	S	S	I	S	S	S	R	R	I	R	S
GF4	796	S	S	I	S	S	S	S	S	S	S	S
GF4	798	S	S	I	S	S	S	S	S	S	S	S
GF4	799	S	S	I	S	S	S	S	S	S	S	S
GF4	800	S	R	R	R	R	S	S	S	R	R	R
GF4	801	S	S	S	S	S	S	S	S	S	S	S
GF5	802	S	R	R	R	R	S	S	S	R	R	R
GF5	803	S	S	I	S	I	S	S	S	S	I	S
GF5	804	S	S	I	S	S	S	S	S	S	S	S

GF5	805	S	R	R	R	R	S	S	S	R	R	R
GF5	806	S	S	I	S	R	S	S	S	S	S	S
GF5	807	S	R	R	R	R	S	S	S	R	R	R
GF5	808	S	R	R	R	R	S	S	S	R	R	R
GF5	809	R	R	R	R	R	R	R	R	R	R	R
KE1	812	S	S	I	S	S	S	S	S	S	S	S
KE2	814	R	R	R	R	R	R	R	R	R	R	R
KE2	815	S	S	I	S	R	R	S	S	S	R	R
KE2	816	S	S	I	S	S	S	S	S	S	R	R
KE2	817	S	I	R	R	R	R	S	S	I	R	R
KE2	818	S	S	I	S	R	R	S	S	S	R	R
KE2	819	S	S	I	S	S	S	S	S	S	R	R
KE3	823	S	S	S	S	S	S	S	S	S	S	S
KE4	826	S	S	I	S	S	S	S	S	S	S	S
KE4	827	S	S	I	S	S	S	S	S	S	S	S
KE4	828	S	S	I	S	S	S	S	S	S	S	S
KE1	831	S	S	I	S	S	S	S	S	S	S	S
KE1	833	S	S	I	I	S	S	S	S	S	S	S
KE1	835	S	S	I	S	S	S	S	S	S	S	S
KE1	836	S	S	I	I	S	S	S	S	S	S	S
KE2	837	S	S	I	S	S	S	S	S	S	R	R
KE2	838	S	S	I	S	S	S	S	S	S	R	R
KE2	839	S	S	I	S	S	S	S	S	S	R	R
KE2	840	S	R	R	R	R	R	R	R	R	R	R
KE2	842	S	S	I	S	S	R	S	S	S	R	R
KE3	843	S	S	I	S	S	R	S	S	S	R	R
KE3	844	S	S	I	S	S	S	S	S	S	S	S
KE3	845	S	S	R	R	R	R	S	S	R	R	R
KE4	851	S	S	I	S	S	S	S	S	S	S	S
KE4	852	S	S	I	S	S	S	S	S	R	S	S
KE4	854	S	S	I	S	S	S	S	S	S	S	S
KE4	856	S	S	I	S	S	S	S	S	S	S	S
GF1	858	S	S	S	S	S	S	S	S	S	S	S
GF2	861	S	S	S	S	S	S	S	S	S	S	S
GF4	870	S	S	S	S	S	S	S	S	S	R	S
GF4	872	S	S	S	S	S	S	S	S	S	R	R
GF4	875	S	S	S	S	S	S	S	S	S	S	S
GF4	878	S	S	S	S	S	S	S	S	S	R	R
GF4	880	S	S	S	S	S	S	S	S	S	R	R
GF4	881	S	S	S	S	S	S	S	S	S	R	R
GF5	885	S	S	S	S	S	S	R	R	I	R	S
GF5	887	S	S	I	S	S	S	S	S	S	S	S
GF5	888	S	S	S	S	S	S	S	S	S	S	S

GF5	890	S	S	I	S	S	S	R	R	S	S	S
TY1	892	S	S	S	S	S	S	S	S	S	S	S
TY1	893	S	R	R	R	R	S	S	S	R	R	R
TY1	894	S	S	I	S	S	S	S	S	S	R	R
TY2	896	S	S	I	S	S	S	R	R	S	R	S
TY2	897	S	S	I	S	S	S	S	S	S	R	R
TY2	898	S	S	I	S	S	S	R	R	I	R	R
TY2	899	S	S	S	S	S	S	I	S	S	S	S
TY2	900	S	S	S	S	S	S	I	S	S	S	S
TY2	901	S	S	I	S	S	S	R	R	S	I	S
KE1	906	S	S	S	S	S	S	S	S	S	S	S
KE2	909	S	S	S	S	S	S	S	S	S	R	S
KE2	911	S	S	I	R	S	R	S	S	S	S	R
KE3	913	S	S	S	S	S	S	S	S	S	I	S
KE3	914	S	S	S	S	I	S	S	S	S	S	S
KE4	916	S	S	I	S	S	S	S	S	S	S	S
KE5	918	S	S	I	R	S	R	S	S	S	S	R
KE5	919	S	S	S	S	S	S	S	S	I	S	S
GF1	920	S	S	I	S	S	S	S	S	S	S	S
GF1	922	S	S	S	S	S	S	S	S	S	S	S
GF1	923	S	S	I	S	S	S	S	S	S	S	S
GF1	924	S	S	I	S	S	S	S	S	S	S	S
GF2	928	S	S	S	S	S	S	S	S	S	S	S
GF3	930	S	S	S	S	S	S	S	S	S	I	S
GF4	931	S	S	R	I	S	R	S	S	S	S	R
GF4	932	S	S	S	S	S	S	S	S	S	R	S
GF4	933	S	S	S	S	S	S	S	S	S	R	R
GF4	934	S	S	R	S	S	S	S	S	S	R	R
GF4	935	S	S	S	S	S	S	S	S	S	R	R
GF5	936	S	S	S	S	S	S	S	S	S	S	S
GF5	937	S	S	I	S	S	S	S	S	S	S	S
GF5	938	S	S	I	S	S	S	S	S	S	S	S
TY1	940	S	S	I	S	S	S	S	S	S	S	S
TY2	944	S	S	I	S	S	S	S	S	S	I	S
TY2	945	S	S	I	S	S	S	S	S	I	S	S
TY3	949	S	S	I	S	S	S	S	S	S	I	S
TY4	954	S	S	I	S	S	S	S	S	I	S	S
TY4	955	S	S	S	S	S	S	S	S	S	I	S
TY5	956	S	S	R	I	I	S	S	S	I	I	S
TY5	957	S	S	R	I	I	S	S	S	I	R	S
TY5	958	S	S	R	I	I	S	S	S	R	I	S
GF4	1019	S	R	R	R	I	S	R	R	R	R	R
GF4	1020	S	R	R	R	I	S	R	R	R	R	R

GF4	1022	S	I	I	R	I	S	S	S	S	S	S
GF5	1023	S	S	R	R	S	S	S	S	I	S	S
GF5	1024	S	R	R	R	S	R	S	S	I	S	R
GF5	1025	S	S	R	R	S	R	S	S	S	S	R
GF5	1026	S	I	R	R	R	R	S	S	R	R	R
TY1	1027	S	R	R	R	R	R	S	S	R	R	R
TY1	1028	S	R	R	R	R	R	S	S	R	R	R
TY2	1031	S	S	R	I	S	S	S	S	I	I	S
TY2	1032	S	S	R	I	I	S	S	S	R	R	R
TY3	1035	S	R	R	I	R	R	S	S	R	R	R
TY4	1041	S	S	R	I	I	S	S	S	I	I	S
TY4	1042	S	S	I	I	S	S	S	S	S	I	S
TY5	1045	S	S	I	I	S	S	S	S	S	S	S
TY5	1046	S	S	I	I	S	S	S	S	S	I	S
KE1	1047	S	S	I	S	S	S	S	S	S	I	S
KE1	1049	S	S	I	S	S	S	S	S	S	S	S
KE2	1053	S	S	I	S	S	S	S	S	S	S	S
KE2	1054	S	S	I	I	S	S	S	S	I	S	S
KE3	1055	S	S	I	I	S	S	S	S	I	S	S
KE3	1056	S	S	I	I	S	S	S	S	S	S	S
KE3	1057	S	S	I	I	S	S	S	S	I	S	S
KE3	1058	S	S	I	I	S	S	S	S	S	S	S
KE4	1059	S	S	R	I	I	S	S	S	I	S	S
KE4	1060	S	S	I	S	I	S	S	S	I	I	S
KE4	1061	S	S	I	S	S	S	S	S	S	S	S
KE4	1062	S	S	I	S	S	S	S	S	I	S	S
KE5	1063	S	S	I	S	S	S	S	S	S	S	S
KE5	1064	S	S	I	S	S	S	S	S	I	S	S
KE5	1065	S	S	I	I	S	S	S	S	S	S	S
KE5	1066	S	S	I	S	S	S	S	S	S	S	S

Table S2: Screening of *A. baumannii* that exhibited phenotypic resistance for antibiotics resistant genes

Antimicrobial Family	Primer Name	PCR primer sequence (5 '–3 ')	Amplicon size (bp)	Gene name	PCR cycling condition	Reference
Aminoglycosides	aph(3)-Ia (*aphA1*)	F: ATGGGCTCGCGATAATGTC<br>R: CTCACCGAGGCAGTTCCAT	600	*apHA1*	Initial denaturation step: 94°C for 5 min, followed by 30 cycles of 94°C for 30 s, annealing step: 50°C for 30 s and 72°C for 1.5 min and final elongation at 72°C for 5 min	[1]
	aph(3)-IIa (*aphA2*)	F: GAACAAGATGGATTGCACGC<br>R: GCTCTTCAGCAATATCACGG	510	*apHA2*	Initial denaturation step: 94°C for 5 min, followed by 30 cycles of 94°C for 30 s, annealing step: 50°C for 30 s and 72°C for 1.5 min and final elongation at 72°C for 5 min	[1]
Beta-lactamases	MultiTSO-T	F: CATTTCCGTGTCGCCCTTATTC<br>R: CGTTCATCCATAGTTGCCTGAC	800	bla_{TEM}	Initial denaturation step: 94°C for 10 min, followed by 30 cycles of 94°C for 40 s, annealing step: 60°C for 40 s and 72°C for 1 min; and a final elongation step at 72°C for 7 min	[2]
	MultiTSO-S	F: AGCCGCTTGAGCAAATTAAAC<br>R: ATCCCGCAGATAAATCACCAC	713	bla_{SHV}	Initial denaturation step: 94°C for 10 min, followed by 30 cycles of 94°C for 40 s, annealing step: 60°C for 40 s and 72°C for 1 min; and final elongation step at 72°C for 7 min	[2]
	MultiCTXMGp 1	F: TTAGGAARTGTGCCGCTGYA<br>R: CGATATCGTTGGTGGTRCCAT	688	$bla_{CTX-M(1)}$	Initial denaturation step: 94°C for 10 min, followed by 30 cycles of 94°C for 40 s, annealing step: 60°C for 40 s and 72°C for 1 min; and final elongation step at 72°C for 7 min	[2]

	MultiCTXMGp 2	F: CGTTAACGGCACGATGAC<br>R: CGATATCGTTGGTGGTRCCAT	404	$bla_{CTX-M(2)}$	Initial denaturation step: 94°C for 10 min, followed by 30 cycles of 94°C for 40 s, annealing step: 60°C for 40 s and 72°C for 1 min; and final elongation step at 72°C for 7 min	[2]
	MultiCTXMGp 9	F: TCAAGCCTGCCGATCTGGT<br>R: TGATTCTCGCCGCTGAAG	561	$bla_{CTX-M(9)}$	Initial denaturation step: 94°C for 10 min, followed by 30 cycles of 94°C for 40 s, annealing step: 60°C for 40 s and 72°C for 1 min; and final elongation step at 72°C for 7 min	[2]
	CTX-Mg8/25	F: AACRCRCAGACGCTCTAC<br>R: TCGAGCCGGAASGTGTYAT	326	$bla_{CTX-M-8/-25}$	Initial denaturation step: 94°C for 10 min, followed by 30 cycles of 94°C for 40 s, annealing step: 60°C for 40 s and 72°C for 1 min; and final elongation step at 72°C for 7 min	[2]
	MultiGES	F: AGTCGGCTAGACCGGAAAG<br>R: TTTGTCCGTGCTCAGGAT	399	bla_{GES}	Initial denaturation step: 94°C for 10 min, followed by 30 cycles of 94°C for 40 s, annealing step: 57°C for 40 s and 72°C for 1 min; and final elongation step at 72°C for 7 min	[2]
	MultiOXA-48<br><br><br><br><br>OXA-51<br><br>OXA-23<br><br>OXA-40<br><br>OXA-58	F: GCTTGATCGCCCTCGATT<br>R: GATTTGCTCCGTGGCCGAAA<br><br><br><br>F: TAATGCTTTGATCGGCCTTG<br>R: TGGATTGCACTTCATCTTGG<br>F: GATCGGATTGGAGAACCAGA<br>R: ATTCTTGACCGCATTTCCAT<br>F: GGTTGTTGGCCCCCTTAAA<br>R: AGTTGAGCGAAAAGGGGATT<br>F: AAGTATTGGGGCTTGTGCTG<br>R: CCCCTCTGCGCTCTACATAC	281<br><br><br><br><br>353<br><br>501<br><br>246<br><br>599	$bla_{OXA-48-like}$<br><br><br><br><br>$bla_{OXA-51-like}$<br><br>$bla_{OXA-23-like}$<br><br>$bla_{OXA-40-like}$<br><br>$bla_{OXA-58-like}$	Initial denaturation step: 94°C for 10 min, followed by 30 cycles of 94°C for 40 s, annealing step: 57°C for 40 s and 72°C for 1 min; and final elongation step at 72°C for 7 mins<br><br>Initial denaturation step: 95°C for 1 min, followed by 30 cycles of 95°C for 15 s, annealing step: 52°C for 15 s and 72°C for 10 s; and final elongation step at 72°C for 10 mins	[2]<br><br><br><br>[3]

	MultiPER	F: GCTCCGATAATGAAAGCGT<br>R: TTCGGCTTGACTCGGCTGA	520	*bla*PER	Initial denaturation step: 94°C for 10 mins, followed by 30 cycles of 94°C for 40 s, annealing step: 60°C for 40 s and 72°C for 1 min; and final elongation step at 72°C for 7 mins	[2]
	MultiVEB	F: CATTTCCCGATGCAAAGCGT<br>R: CGAAGTTTCTTTGGACTCTG	648	*bla*VEB	Initial denaturation step: 94°C for 10 min, followed by 30 cycles of 94°C for 40 s, annealing step: 60°C for 40 s and 72°C for 1 min; and final elongation step at 72°C for 7 min	[2]
	MultiIMP	F: TTGACACTCCATTTACDG<br>R: GATYGAGAATTAAGCCACYCT	139	*bla*IMP	Initial denaturation step: 94°C for 10 min, followed by 30 cycles of 94°C for 40 s, annealing step: 55°C for 40 s and 72°C for 1 min; and final elongation step at 72°C for 7 min	[2]
	MultiVIM	F: GATGGTGTTTGGTCGCATA<br>R: CGAATGCGCAGCACCAG	390	*bla*VIM	Initial denaturation step: 94°C for 10 min, followed by 30 cycles of 94°C for 40 s, annealing step: 55°C for 40 s and 72°C for 1 min; and final elongation step at 72°C for 7 min	[2]
	MultiKPC	F: CATTCAAGGGCTTTCTTGCTGC<br>R: ACGACGGCATAGTCATTTGC	700	*bla*KPC	Initial denaturation step: 94°C for 10 min, followed by 30 cycles of 94°C for 40 s, annealing step: 55°C for 40 s and 72°C for 1 min; and final elongation step at 72°C for 7 min	[2]
Fluoroquinolone s	QnrA	F: AGAGGATTTCTCACGCCAGG<br>R: TGCCAGGCACAGATCTTGAC	580	*qnrA*	Initial denaturation step: 95°C for 15 min, followed by 32 cycles of 95°C for 1 min, annealing step: 55°C for 1 min, and 72°C for 5 min and final longation step at 72°C for 10 min	[4]

	QnrB	F: GGMATHGAAATTCGCCACTG<br>R: TTTGCYGYYCGCCAGTCGAA	264	*qnrB*	Initial denaturation step: 95°C for 15 min, followed by 32 cycles of 95°C for 1 min, annealing step: 55°C for 1 min, and 72°C for 5 min and final elongation step at 72°C for 10 min	[4]
	QnrC	F: GGGTTGTACATTTATTGAATCG<br>R: CACCTACCCATTTATTTTCA	307	*qnrC*	Initial denaturation step: 95°C for 15 min, followed by 32 cycles of 95°C for 1 min, annealing step: 55°C for 1 min, and 72°C for 5 min and final elongation step at 72°C for 10 min	[5]
	QnrD	F: CGAGATCAATTTACGGGGAATA<br>R: AACAAGCTGAAGCGCCTG	533	*qnrD*	Initial denaturation step: 95°C for 15 min, followed by 32 cycles of 95°C for 1 min, annealing step: 55°C for 1 min, and 72°C for 5 min and final elongation step at 72°C for 10 min	[4]
	QnrS	F: ACGACATTCGTCAACTGCAA<br>R: TAAATTGGCACCCTGTAGGC	417	*qnrS*	Initial denaturation step: 95°C for 15 min, followed by 32 cycles of 95°C for 1 min, annealing step: 55°C for 1 min, and 72°C for 5 min and final elongation step at 72°C for 10 min	[5]
Sulfonamides	sul1	F: CGGCGTGGGCTACCTGAACG<br>R: GCCGATCGCGTGAAGTTCCG	433	*sul1*	Initial denaturation step: 94°C for 5 min, followed by 30 cycles of 94°C for 30 s, annealing step: 65°C for 30 s, 72°C for 2 min, and final extension step at 72°C for 10 min	[6]
	sul2	F: GCGCTCAAGGCAGATGGCATT<br>R: GCGTTTGATACCGGCACCCGT	293	*sul2*	Initial denaturation step: 94°C for 5 min, followed by 30 cycles of 94°C for 30 s, annealing step: 65°C for 30 s, 72°C for 2 min, and final extension step at 72°C for 10 min	[6]

Tetracyclines	TetA	F: GCTACATCCTGCTTGCCTTC<br>R: CATAGATCGCCGTGAAGAGG	210	*tet*(A)	Initial denaturation step: 94°C for 5 min, followed by 30 cycles of 94°C for 30 s, annealing step: 60°C for 30 s, 72°C for 2 min, and final extension step at 72°C for 10 min	[6]
	TetB	F: TTG GTT AGG GGC AAG TTT TG<br>R: GTA ATG GGC CAA TAA CAC CG	659	*tet*(B)	Initial denaturation step: 94°C for 5 min, followed by 35 cycles of 94°C for 1 min, annealing step: 55°C for 1 min, 72°C for 2 min and final elongation step: 72°C for 1·5 min	[7]
	TetC	F: CTTGAGAGCCTTCAACCCAG<br>R: ATGGTCGTCATCTACCTGCC	418	*tet*(C)	Initial denaturation step: 94°C for 5 min, followed by 30 cycles of 94°C for 30 s, annealing step: 60°C for 30 s, 72°C for 2 min, and final extension step at 72°C for 10 min	[6]
	TetL	F: TCG TTA GCG TGC TGT CAT TC<br>R: GTA TCC CAC CAA TGT AGC CG	267	*tet*(L)	Initial denaturation step: 94°C for 5 min, followed by 35 cycles of 94°C for 1 min, annealing step: 55°C for 1 min and final elongation step: 72°C for 1·5 min	[7]
	TetM	F: GTG GAC AAA GGT ACA ACG AG<br>R: CGG TAA AGT TCG TCA CAC AC	406	*tet*(M)	Initial denaturation step: 94°C for 5 min, followed by 35 cycles of 94°C for 1 min, annealing step: 55°C for 1 min and final elongation step: 72°C for 1·5 min	[7]
	TetO	F: ACGGARAGTTTATTGTATACC<br>R: TGGCGTATCTATAATGTTGAC	171	*tet*(O)	Initial denaturation step: 94°C for 5 min, followed by 35 cycles of 94°C for 1 min, annealing step: 55°C for 1 min and final elongation step: 72°C for 1·5 min	[6]

1. Maynard, C.; Bekal, S.; Sanschagrin, F.; Levesque, R.C.; Brousseau, R.; Masson, L.; Lariviere, S.; Harel, J. Heterogeneity among virulence and antimicrobial resistance gene profiles of extraintestinal *Escherichia coli* isolates of animal and human origin. *J. Clin. Microbiol.* **2004**, *42*, 5444–5452.
2. Dallenne, C.; Da Costa, A.; Decre, D.; Favier, C.; Arlet, G. Development of a set of multiplex PCR assays for the detection of genes encoding important b-lactamases in Enterobacteriaceae. *J. Antimicrob. Chemother.* **2010**, *65*, 490–495.
3. Abouelfetouh, A.; Torky, A.S. Aboulmagd, E. Phenotypic and genotypic characterization of carbapenem-resistant *Acinetobacter baumannii* isolates from Egypt. *Antimicrob. Resist. Infection Cont*. ***2019****, 8, 185*.
4. El-Badawy, M.F.; Tawakol, W.M.; El-Far, S.W.; Maghrabi, I.A.; Al-Ghamdi, S.A.; Mansy, M.S.; Ashour, M.S.; Shohaye, M.M. Molecular Identification of Aminoglycoside-Modifying Enzymes and Plasmid-Mediated Quinolone Resistance Genes among Klebsiella pneumoniae Clinical Isolates Recovered from Egyptian Patients. *Int. J. Microbiol.* **2017**, *2017*, 8050432.
5. Pribul, B.R.; Festivo, M.L.; Rodrigues, M.S.; Costa, R.G.; Rodrigues, E.C.d.P.; de Souza, M.M.S.; Rodrigues, D.d.P. Characteristics of Quinolone Resistance in *Salmonella* spp. isolates from the food chain in Brazil. *Front. Microbiol.* **2017**, *8*, 299.
6. Li, L.; Ye, L.; Zhang, S.; Meng, H. Isolation and identification of aerobic bacteria carrying tetracycline and sulfonamide resistance genes obtained from a meat processing plant. *J. Food Sci.* **2016**, *81*, M1480–M1484.
7. Ng, L.K.; Martin, I.; Alfa, M.; Mulvey, M. Multiplex PCR for the detection of tetracycline resistant genes. *Mol. Cell. Probes* **2001**, *15*, 209–215.
